# Supplementary material for: Efficient Synchronization of Dipolarly Coupled Vortex-Based Spin Transfer Nano-Oscillators
Source: Sci Rep. 2015 Nov 26;5:17039. doi: 10.1038/srep17039 (PMC4660301; doi:10.1038/srep17039)
Supplement: Supplementary Information [file srep17039-s1.pdf]

Efficient Synchronization of Dipolarly Coupled Vortex-Based Spin Transfer Nano-Oscillators

Nicolas Locatelli[1,*], Abbass Hamadeh[2], Flavio Abreu Araujo[3], Anatoly D. Belanovsky[4,5], Petr N. Skirdkov[4,5], Romain Lebrun[1], Vladimir V. Naletov[1,2,6], Konstantin A. Zvezdin[4,5,7], Manuel Muñoz[8], Julie Grollier[1], Olivier Klein[2,+], Vincent Cros[1,], and Grégoire de Loubens[2]**

[1]Unité Mixte de Physique CNRS/Thales and Université Paris Sud, F91767 Palaiseau, France
[2]Service de Physique de l'Etat Condensé, CNRS UMR 3680, CEA Saclay, F91191 Gif-sur-Yvette, France
[3]Institute of Condensed Matter and Nanosciences, Université catholique de Louvain, BE-1348 Louvain-la-Neuve, Belgium
[4]Moscow Institute of Physics and Technology (State University), Institutskiy per. 9, 141700 Dolgoprudny, Russia
[5]A. M. Prokhorov General Physics Institute, RAS, Vavilova 38, Moscow, Russia
[6]Institute of Physics, Kazan Federal University, Kazan 420008, Russian Federation
[7]Istituto P.M. srl, Via Grassi 4, Torino, Italy
[8]Instituto de Microelectrónica de Madrid-IMM (CNM-CSIC), Isaac Newton 8-PTM, 28760 Tres Cantos, Madrid, Spain
[*]nicolas.locatelli@u-psud.fr, Present address: Institut d'Electronique Fondamentale, Université Paris-Sud, UMR CNRS 8622, F91405 Orsay, France
[+]Present address: INAC-SPINTEC, CEA/CNRS and Univ. Grenoble Alpes, 38000 Grenoble, France
[**]vincent.cros@thalesgroup.com

ABSTRACT

Due to their nonlinear properties, spin transfer nano-oscillators can easily adapt their frequency to external stimuli. This makes them interesting model systems to study the effects of synchronization and brings some opportunities to improve their microwave characteristics in view of their applications in information and communication technologies and to design innovative computing architectures. So far, mutual synchronization of spin transfer nano-oscillators through propagating spin-waves and exchange coupling in a common magnetic layer has been demonstrated. Here we show that the dipolar interaction is also an efficient mechanism to synchronize neighbouring oscillators. We experimentally study a pair of vortex-based spin-transfer nano-oscillators, in which mutual synchronization can be achieved despite a significant frequency mismatch between oscillators. Importantly, the coupling efficiency is controlled by the magnetic configuration of the vortices, as confirmed by an analytical model highlighting the physics at play in the synchronization process as well as by micromagnetic simulations.

Supplementary materials

The vortex magnetization and the Thiele equation

In the absence of crystalline anisotropy and for adequate dimensions, the remanent magnetic state of a cylinder shaped nanomagnet is a magnetic vortex.[1] The magnetization lies in the plane, winding around the centre of the magnet where it pops out of the plane in a small region called the vortex core. The vortex state is characterized by two parameters, namely the polarity $P = \pm 1$ giving the direction of the out-of-plane magnetization in the core and the chirality $C = \pm 1$ associated to the curling direction of the in-plane magnetization.

The lowest energy mode of a magnetic vortex is the gyrotropic mode, corresponding to a circular displacement of the vortex core around the centre of the ferromagnet.[1] The associated magnetization dynamics can be described through a single collective variable equation, called the Thiele equation:[2–6]

$$\vec{G} \times \dot{\vec{X}} = -\alpha\eta G\dot{\vec{X}} - k(X)\vec{X} + F_{\mathrm{STT}}(\vec{X}) \tag{1}$$

where $\vec{X}$ is the displacement of the vortex core relative to the center of the dot. The gyrotropic term (LHS) is equal to the sum of the effective dissipative and conservative forces acting on the vortex core (RHS).

The LHS terms features the gyrovector $\vec{G} = -PG\hat{z} = -2\pi PL\frac{M_S}{\gamma}\hat{z}$. P is the vortex polarity, L is the layer thickness, M_S is the ferromagnet saturation magnetization, γ is the gyromagnetic ratio.

The RHS terms respectively are :

- The viscous damping force (proportional and opposed to the core speed): $\vec{F} = -\alpha\eta G\dot{\vec{X}}$, accounting for the dissipative phenomena opposing the motion. α is the Gilbert damping parameter of the ferromagnet ($\alpha = 0.01$ for NiFe), and $\eta = \frac{1}{2}\ln\left(\frac{R}{2l_{ex}}\right) - \frac{1}{8}$ is a phenomenological damping coefficient for the vortex .[4,5,7] R is the pillar radius, l_{ex} is the material exchange length ($l_{ex} \simeq 6$nm for NiFe).
- The spring-like confinement force (opposed to the core displacement): $\vec{F} = -k(X)\vec{X}$. $W(\vec{X}) = \frac{1}{2}k(X)X^2$ is the shifted vortex energy, with $k(X) = \omega_0 G\left(1 + a\left(\frac{X}{R}\right)^2\right)$ including both the magneto-static and the Oersted field contributions:[4,8] $\omega_0 = \frac{5}{9\pi}\gamma\mu_0 M_S\frac{L}{R} + C\frac{0.85}{2\pi}\gamma\mu_0 RJ$ (C is the vortex chirality, J is the current density flowing through the pillar).
- A supplementary force added to account for spin-transfer effects: $F_{STT}(\vec{X})$. In the general case of a non-uniform polarizer, the spin transfer force direction depends on the core polarity sign, and can be described by the general expression: $\vec{F}_{STT}(\vec{X}) = P\lambda(X,J)\hat{z}\times\vec{X}$, where $\lambda(X,J)$ expresses the spin transfer torque efficiency.[3–6,9,10]

Using complex coordinates, the Thiele equation writes:

$$(\mathrm{i}PG + \alpha\eta G)\dot{\boldsymbol{X}} = -(k(X) - \mathrm{i}P\lambda(X,J))\boldsymbol{X} \tag{2}$$

where $\boldsymbol{X} = X_i e^{\mathrm{i}\varphi}$ is the complex coordinate of the core position.

$$\dot{\boldsymbol{X}} = -\left(\frac{\alpha\eta k(X)}{G} - \frac{\lambda(X,J)}{G}\right)\boldsymbol{X} + \mathrm{i}P\left(\frac{k(X)}{G} + \frac{\alpha\eta\lambda(X,J)}{G}\right)\boldsymbol{X} \tag{3}$$

$$\begin{cases} \dot{X} = \left[\frac{\lambda(X,J)}{G} - \frac{\alpha\eta k(X)}{G}\right]X & \text{(4a)} \\ \dot{\varphi} = P\left[\frac{k(X)}{G} + \frac{\alpha\eta\lambda(X,J)}{G}\right] & \text{(4b)} \end{cases}$$

While equation 4a determines the equilibrium orbit radius for the core gyration, equation 4b determines the frequency of the motion. An important conclusion drawn from this second equation is that the sense of gyration of the vortex core is directly related to its polarity sign P.[1]

Derivation of the synchronization equation

Starting with the two coupled Thiele equations:

$$\begin{cases} \vec{G}_1\times\dot{\vec{X}}_1 = -D_1(X_1)\dot{\vec{X}}_1 - k_1(X_1)\vec{X}_1 + \vec{F}_{STT}(\vec{X}_1) + \vec{F}_{int}^{2\to1}(\vec{X}_2) & \text{(5a)} \\ \vec{G}_2\times\dot{\vec{X}}_2 = -D_2(X_2)\dot{\vec{X}}_2 - k_2(X_2)\vec{X}_2 + \vec{F}_{STT}(\vec{X}_2) + \vec{F}_{int}^{1\to2}(\vec{X}_1) & \text{(5b)} \end{cases}$$

The interaction force is expressed as : $\vec{F}_{int}^{j\to i} = -\frac{\partial W_{int}}{\partial \vec{X}_i}$, and the spin transfer force is described by the general expression: $\vec{F}_{STT}(\vec{X}_i) = P_i\lambda(X_i,J_i)\hat{z}\times\vec{X}_i$ where J_i. By using complex coordinates, these equations can then be simplified as :

$$\begin{cases} (\mathrm{i}P_1G_1 + \alpha\eta_1G_1)\dot{\boldsymbol{X}}_1 = -(k_1(X_1) - \mathrm{i}P_1\lambda(X_1,J_1))\boldsymbol{X}_1 - C_1C_2(\mu_{(+)}\boldsymbol{X}_2^* - \mu_{(-)}\boldsymbol{X}_2) & \text{(6a)} \\ (\mathrm{i}P_2G_2 + \alpha\eta_2G_2)\dot{\boldsymbol{X}}_2 = -(k_2(X_2) - \mathrm{i}P_2\lambda(X_2,J_1))\boldsymbol{X}_2 - C_1C_2(\mu_{(+)}\boldsymbol{X}_1^* - \mu_{(-)}\boldsymbol{X}_1) & \text{(6b)} \end{cases}$$

where $\boldsymbol{X}_i = X_i e^{\mathrm{i}\varphi_i}$ ($i \in \{1,2\}$) are the complex coordinates of the core positions and $\boldsymbol{X}_{1,2}{}^* = X_i e^{-\mathrm{i}\varphi_i}$ are their complex conjugates.

$$
\begin{cases}
\dot{\boldsymbol{X}}_1 = -\left(\frac{\alpha\eta_1 k_1(X_1)}{G_1} - \frac{\lambda(X_1,J_1)}{G_1}\right)\boldsymbol{X}_1 + \mathrm{i}P_1\left(\frac{k_1(X_1)}{G_1} + \frac{\alpha\eta_1\lambda(X_1,J_1)}{G_1}\right)\boldsymbol{X}_1 \\
\qquad - \frac{\alpha\eta(X_1)C_1C_2}{G_1}\left(\mu_{(+)}\boldsymbol{X}_2^* - \mu_{(-)}\boldsymbol{X}_2\right) + \mathrm{i}P_1\frac{C_1C_2}{G_1}\left(\mu_{(+)}\boldsymbol{X}_2^* - \mu_{(-)}\boldsymbol{X}_2\right) & (7a) \\
\dot{\boldsymbol{X}}_2 = -\left(\frac{\alpha\eta_2 k_2(X_2)}{G_2} - \frac{\lambda(X_2,J_2)}{G_2}\right)\boldsymbol{X}_2 + \mathrm{i}P_2\left(\frac{k_2(X_2)}{G_2} + \frac{\alpha\eta_2\lambda(X_2,J_2)}{G_2}\right)\boldsymbol{X}_2 \\
\qquad - \frac{\alpha\eta(X_2)C_1C_2}{G_2}\left(\mu_{(+)}\boldsymbol{X}_1^* - \mu_{(-)}\boldsymbol{X}_1\right) + \mathrm{i}P_2\frac{C_1C_2}{G_2}\left(\mu_{(+)}\boldsymbol{X}_1^* - \mu_{(-)}\boldsymbol{X}_1\right) & (7b)
\end{cases}
$$

$$
\begin{cases}
\frac{\dot{X}_i}{X_i} = \left[\frac{\lambda(X_i,J_i)}{G_i} - \frac{\alpha\eta_i k_i(X_i)}{G_i}\right] - \frac{\alpha\eta_i C_1C_2}{G_i} Re\left[\mu_{(+)}\frac{\boldsymbol{X}_j^*}{\boldsymbol{X}_i} - \mu_{(-)}\frac{\boldsymbol{X}_j}{\boldsymbol{X}_i}\right] \\
\qquad - P_i\frac{C_1C_2}{G_i} Im\left[\mu_{(+)}\frac{\boldsymbol{X}_j^*}{\boldsymbol{X}_i} - \mu_{(-)}\frac{\boldsymbol{X}_j}{\boldsymbol{X}_i}\right] & (8a) \\
\dot{\varphi}_i = P_i\left[\frac{k_i(X_i)}{G_i} + \frac{\alpha\eta_i\lambda(X_i,J_i)}{G_i}\right] - \frac{\alpha\eta_i C_1C_2}{G_i} Im\left[\mu_{(+)}\frac{\boldsymbol{X}_j^*}{\boldsymbol{X}_i} - \mu_{(-)}\frac{\boldsymbol{X}_j}{\boldsymbol{X}_i}\right] \\
\qquad + P_i\frac{C_1C_2}{G_i} Re\left[\mu_{(+)}\frac{\boldsymbol{X}_j^*}{\boldsymbol{X}_i} - \mu_{(-)}\frac{\boldsymbol{X}_j}{\boldsymbol{X}_i}\right] & (8b)
\end{cases}
$$

In the absence of coupling, the auto-oscillator gyration radius is set by the relationship: $\lambda(X_i^0, J_i) = \alpha\eta_i k_i(X_i^0)$, while the frequency is given by: $\dot{\varphi}_i = P_i\omega_i = P_i\left(\frac{k_i(X_i)}{G_i} + \frac{\alpha\eta_i\lambda(X_i,J_i)}{G_i}\right)$. In the following, we will consider deviations of the gyration radii around their equilibrium values through the oscillations power: $p_i = \left(\frac{X_i}{R_i}\right)^2 = p_{i_0} + \delta p_i$, and the subsequent deviations of the instantaneous frequencies: $P_i\dot{\varphi}_i = \omega_i + N_i\delta p_i$, and dissipative forces: $\frac{\alpha\eta_i k_i(X_i)}{G_i} - \frac{\lambda(X_i,J_i)}{G_i} = 2\Gamma_{p_i}\delta p_i$. The terms associated with dipolar coupling are then considered as perturbations of the equilibrium. Inside these terms, the approximation $X_1/X_2 \simeq X_1^0/X_2^0 \simeq 1$ can be used. So that we obtain:

$$
\begin{cases}
\dot{\delta p_i} = -2\Gamma_{p_i}\delta p_i - \frac{2\alpha\eta_i C_1C_2}{G_i}p_{i_0}\left(\mu_{(+)}\cos(\varphi_j+\varphi_i) - \mu_{(-)}\cos(\varphi_j-\varphi_i)\right) \\
\qquad + \frac{2P_iC_1C_2}{G_i}p_{i_0}\left(\mu_{(+)}\sin(\varphi_j+\varphi_i) + \mu_{(-)}\sin(\varphi_j-\varphi_i)\right) & (9a) \\
P_i\dot{\varphi}_i = \omega_i + N_i\delta p_i + P_i\frac{\alpha\eta C_1C_2}{G_i}\left(\mu_{(+)}\sin(\varphi_j+\varphi_i) + \mu_{(-)}\sin(\varphi_j-\varphi_i)\right) \\
\qquad + \frac{C_1C_2}{G_i}\left(\mu_{(+)}\cos(\varphi_j+\varphi_i) - \mu_{(-)}\cos(\varphi_j-\varphi_i)\right) & (9b)
\end{cases}
$$

From these equations, it appears that the dipolar coupling action comes with two different time rates: $(\omega_1+\omega_2)$ and $|\omega_1-\omega_2|$. Phases and radii variations at the frequency $(\omega_1+\omega_2)$ will be averaged out in the synchronization process, so that only the low frequency contributions at frequency $|\omega_1-\omega_2|$ should be considered. At this step, we must note that the relative polarities signs will decide which terms should be cancelled or kept.

For the sake of simplicity, we will continue the demonstration for the case $P_1 = P_2 = +1$. In this case, terms associated with $\mu_{(+)}$ are cancelled out:

$$
\begin{cases}
\dot{\delta p_i} = -2\Gamma_{p_i}\delta p_i + 2C_1C_2\frac{\mu_{(-)}}{G_i}p_{i_0}\left(\alpha\eta_i\cos(\varphi_j-\varphi_i) + \sin(\varphi_j-\varphi_i)\right) & (10a) \\
\dot{\varphi}_i = \omega_i + N_i\delta p_i + C_1C_2\frac{\mu_{(-)}}{G_i}\left(\alpha\eta_i\sin(\varphi_j-\varphi_i) - \cos(\varphi_j-\varphi_i)\right) & (10b)
\end{cases}
$$

For the slow variations of the gyration radii, we can consider that an equilibrium is permanently reached ($\dot{\delta p_i} = 0$):

$$
\delta p_i = \frac{p_{i_0}}{\Gamma_{p_i}}C_1C_2\frac{\mu_{(-)}}{G_i}\left(\alpha\eta_i\cos(\varphi_j-\varphi_i) + \sin(\varphi_j-\varphi_i)\right) \tag{11}
$$

So that the slow dynamics of the phases are given by :

$$\dot{\varphi}_i = \omega_i + \frac{N_i p_{i_0}}{\Gamma_{p_i}} C_1 C_2 \frac{\mu_{(-)}}{G_i} \left(\alpha\eta_i \cos(\varphi_j - \varphi_i) + \sin(\varphi_j - \varphi_i)\right) \tag{12}$$

$$+ C_1 C_2 \frac{\mu_{(-)}}{G_i} \left(\alpha\eta_i \sin(\varphi_j - \varphi_i) - \cos(\varphi_j - \varphi_i)\right) \tag{13}$$

$$\dot{\varphi}_i = \omega_i + C_1 C_2 \frac{\mu_{(-)}}{G_i} \left[(\nu_i + \alpha\eta_i) \sin(\varphi_j - \varphi_i) + (\alpha\eta_i \nu_i - 1) \cos(\varphi_j - \varphi_i)\right] \tag{14}$$

where $\nu_i = \frac{N_i p_{i_0}}{\Gamma_{p_i}}$

Given the small applied field and the small discrepancies between the two pillars, and to capture the dominant mechanisms responsible for synchronization, we will use the following approximations: $G_1 \simeq G_2$, $N_1 \simeq N_2$, $\nu_1 \simeq \nu_2$, $\eta_1 \simeq \eta_2$ and suppose that the two auto-oscillators only differs through their frequencies $\omega_1 \neq \omega_2$. We can finally obtain the differential equation governing the dynamics of the phase difference $\Psi = \varphi_1 - \varphi_2$:

$$\dot{\Psi} = (\omega_1 - \omega_2) - 2 C_1 C_2 \frac{\mu_{(-)}}{G} (\nu + \alpha\eta) \sin(\Psi) \tag{15}$$

In case of opposite polarities ($P_1 = +1, P_2 = -1$), these equations become:

$$\begin{cases} \dot{\delta p_i} = -2\Gamma_{p_i} \delta p_i + 2 C_1 C_2 \frac{\mu_{(+)}}{G_i} p_{i_0} \left(-\alpha\eta_i \cos(\varphi_j + \varphi_i) + P_i \sin(\varphi_j + \varphi_i)\right) & \text{(16a)} \\ P_i \dot{\varphi}_i = \omega_i + N_i \delta p_i + C_1 C_2 \frac{\mu_{(+)}}{G_i} \left(P_i \alpha\eta_i \sin(\varphi_j + \varphi_i) + \cos(\varphi_j + \varphi_i)\right) & \text{(16b)} \end{cases}$$

$$\delta p_i = \frac{p_{i_0}}{\Gamma_{p_i}} C_1 C_2 \frac{\mu_{(+)}}{G_i} \left(-\alpha\eta_i \cos(\varphi_j + \varphi_i) + P_i \sin(\varphi_j + \varphi_i)\right) \tag{17}$$

$$P_i \dot{\varphi}_i = \omega_i + \frac{N_i p_{i_0}}{\Gamma_{p_i}} C_1 C_2 \frac{\mu_{(+)}}{G_i} \left(-\alpha\eta_i \cos(\varphi_j + \varphi_i) + P_i \sin(\varphi_j + \varphi_i)\right) \tag{18}$$

$$+ C_1 C_2 \frac{\mu_{(+)}}{G_i} \left(P_i \alpha\eta_i \sin(\varphi_j + \varphi_i) + \cos(\varphi_j + \varphi_i)\right) \tag{19}$$

$$P_i \dot{\varphi}_i = \omega_i + C_1 C_2 \frac{\mu_{(+)}}{G_i} \left[P_i (\nu_i + \alpha\eta_i) \sin(\varphi_j + \varphi_i) + (-\alpha\eta_i \nu_i + 1) \cos(\varphi_j + \varphi_i)\right] \tag{20}$$

$$\begin{cases} \dot{\varphi}_1 = \omega_1 + C_1 C_2 \frac{\mu_{(+)}}{G} \left[(\nu + \alpha\eta) \sin(\varphi_2 + \varphi_1) + (-\alpha\eta\nu + 1) \cos(\varphi_2 + \varphi_1)\right] & \text{(21a)} \\ \dot{\varphi}_2 = -\omega_2 + C_1 C_2 \frac{\mu_{(+)}}{G} \left[(\nu + \alpha\eta) \sin(\varphi_1 + \varphi_2) - (-\alpha\eta\nu + 1) \cos(\varphi_1 + \varphi_2)\right] & \text{(21b)} \end{cases}$$

We can finally obtain the differential equation governing the dynamics of the signed phase difference $\Psi = P_1\varphi_1 + P_2\varphi_2 = \varphi_1 + \varphi_2$:

$$\dot{\Psi} = (\omega_1 - \omega_2) + 2 C_1 C_2 \frac{\mu_{(+)}}{G} (\nu + \alpha\eta) \sin(\Psi) \tag{22}$$

References

1. Guslienko, K. Y. Magnetic vortex state stability, reversal and dynamics in restricted geometries. *J Nanosci. Nanotechno.* **8**, 2745–2760 (2008).
2. Thiele, A. A. Steady-State motion of magnetic domains. *Phys. Rev. Lett.* **30**, 230–233 (1973).
3. Gaididei, Y., Kravchuk, V. P. & Sheka, D. D. Magnetic vortex dynamics induced by an electrical current. *Int. J. Quant. Chem.* **110**, 83–97 (2010).
4. Ivanov, B. A. & Zaspel, C. E. Excitation of spin dynamics by Spin-Polarized current in vortex state magnetic disks. *Phys. Rev. Lett.* **99**, 247208 (2007).
5. Khvalkovskiy, A. V., Grollier, J., Dussaux, A., Zvezdin, K. A. & Cros, V. Vortex oscillations induced by spin-polarized current in a magnetic nanopillar: Analytical versus micromagnetic calculations. *Phys. Rev. B* **80**, 140401 (2009).
6. Mistral, Q. *et al.* Current-Driven vortex oscillations in metallic nanocontacts. *Phys. Rev. Lett.* **100**, 257201 (2008).
7. Dussaux, A. *et al.* Field dependence of spin-transfer-induced vortex dynamics in the nonlinear regime. *Phys. Rev. B* **86**, 014402 (2012).
8. Guslienko, K. Y., Han, X. F., Keavney, D. J., Divan, R. & Bader, S. D. Magnetic vortex core dynamics in cylindrical ferromagnetic dots. *Phys. Rev. Lett.* **96**, 067205 (2006).
9. Khvalkovskiy, A. V. *et al.* Nonuniformity of a planar polarizer for spin-transfer-induced vortex oscillations at zero field. *Appl. Phys. Lett.* **96**, 212507–212507–3 (2010).
10. Sluka, V. *et al.* Quenched slonczewski windmill in spin-torque vortex oscillators. *Phys. Rev. B* **86**, 214422 (2012).
